# Functional and morphological outcome in patients with chronic central serous chorioretinopathy treated by subthreshold micropulse laser

**DOI:** 10.1007/s00417-017-3783-x

**Published:** 2017-08-22

**Authors:** Maciej Gawęcki, Agnieszka Jaszczuk-Maciejewska, Anna Jurska-Jaśko, Andrzej Grzybowski

**Affiliations:** 1 Dobry Wzrok Ophthalmological Clinic, Kliniczna 1B/2, 80-402 Gdańsk, Poland; 2 Chair of Ophthalmology, University of Warmia and Mazury, Warszawska 30, 10-082 Olsztyn, Poland; 3 Department of Ophthalmology, Poznan City Hospital, Poznan, Poland

**Keywords:** subthreshold micropulse laser, central serous chorioretinopathy, subretinal fluid, spectral optical coherence tomography

## Abstract

**Background:**

Chronic central serous chorioretinopathy is a serious therapeutic problem as it may lead to significant visual impairment. The aim of this retrospective study is to evaluate functional and morphological effects, as well as factors influencing visual outcome in patients treated by subthreshold micropulse laser.

**Methods:**

Fifty-one eyes with chronic central serous chorioretinopathy lasting longer than 4 months (18 months on average) underwent up to two sessions of subtreshold micropulse laser treatment. Change in best corrected visual acuity, central retinal thickness, central retinal volume, average central retinal thickness and maximum subretinal fluid height were measured. Relation between the effects of the treatment and the initial retinal morphological and functional parameters was then analyzed.

**Results:**

The total resorption of subretinal fluid was achieved in 36 cases (70.6%). There was, however, only a minor improvement in best corrected visual acuity of approximately one line on the Snellen chart. No correlation was observed between the effects of the treatment and the duration of the symptoms, retinal morphology and initial visual acuity. Younger patients responded better to the therapy.

**Conclusion:**

Subthreshold micropulse laser treatment in chronic serous chorioretinopathy provides good morphological results, however significant improvement of visual acuity is not to be expected.

## Introduction

Central serous chorioretinopathy (CSCR) is a fairly common, well-described clinical entity [1–3]. For the most part, it presents in an acute form, in which symptoms recede spontaneously after a few months. This form of CSCR has a good prognosis and does not impair visual acuity. In its chronic form, however, CSCR poses a real threat to quality of vision, the majority of patients ending up with some form of visual defect, usually a moderate decrease in best corrected visual acuity (BCVA), metamorphopsia or scotoma. A significant decrease in BCVA is noted in a minority of cases, however, for most patients the symptoms of the disease are very alarming and often prevent them from participating in their everyday professional activity [4–7]. It should also be stressed that CSCR normally affects young and active people, for whom even a moderate visual disturbance is significant. Moreover, this clinical entity is largely associated with type A personality, hence the reason why, for some patients, CSCR symptoms are simply unbearable [8, 9].

Treatments for CSCR have been sought for many years, with laser photocoagulation of the leakage point representing a practical solution in selected cases of longer duration [10, 11]. In such cases the leakage point was required to be located at a safe distance from the center of the fovea. Despite observance of this rule, however, some patients have complained of visual scotomas after undergoing such therapy. In addition to this method, practitioners have looked to numerous types of oral medication in search of a resolution for CSCR symptoms (antibiotics, non-steroidal anti-inflammatory drugs, acetazolamide, rifampin, low doses of aspirin, etc.). However, any results they have obtained have not been confirmed in randomized trials [12–16]. Just recently, mineralocorticoid pathway inhibitors have been tested in CSCR treatment with promising results [17–19]. Photodynamic therapy (PDT) is an important form of treatment of chronic CSCR, which is, nevertheless, costly and unavailable in some regions [20–24]. Use of a 689-nm wavelength laser with verteporfin in the PDT procedure was also attempted alone in the treatment of CSCR. The study comparing results of PDT treatment and sole 689 nm laser treatment of CSCR showed that both procedures were equally effective; however, it included cases of relatively short duration of CSCR (17-19 weeks) [25]. The efficacy of anti-VEGF treatment in chronic CSCR is disputable [26–29], with recent data failing to confirm its superiority over PDT or other therapies [30, 31].

In light of these clinical experiences, subthreshold micropulse laser treatment (SMPLT) represents an opportunity for a cheap and effective form of therapy.

The abovementioned type of therapy has been used for treatment of CSCR for the last few years with promising results. In the micropulse mode the energy of the laser is delivered to the tissues in the train of very short repetitive impulses. Effective time of the laser impact is described as duty cycle and for retinal diseases usually set at as low as 5%. The idea of application of subthreshold micropulse laser is stimulation of the RPE to production of antiangiogenic factors without damage to the sensory retina. In consequence, intraretinal or subretinal fluid is easier to be absorbed. Photothermal effect is limited to the RPE only, and due to subthreshold and micropulse modes, is minimal [32–34]. Properly performed SMPLT leaves the sensor retina without any trace, neither visible nor detectable by fundus autofluorescence (FAF) or fluorescein angiography (FA).

This study attempted to analyze results of SMPLT treatment in chronic CSCR, as well as determine factors that could influence functional and morphological outcome. The objective was to find a possible correlation between the effects of SMPLT and the following parameters: age of patient, duration of symptoms, retinal morphology before treatment (retinal thickness and amount of subretinal fluid), and initial visual acuity.

## Material and methods

All procedures performed in this study were in accordance with the ethical standards of the institutional research committee and with the 1964 Helsinki declaration. The study included 51 patients with chronic CSCR, who had been treated with SMPLT at our outpatient ophthalmological clinic during the last 2 years. CSCR was considered chronic when symptoms persisted for longer than 4 months. The 51 patients treated were made up of 17 women (33.3%) and 34 men (66.7%), the age of the patients ranging from 32 to 80 years, with an average of 53.8 years +/- 11.2. The duration of CSCR extended from 4 to 72 months, giving an average of 18.4 months +/- 17.6. Distribution of patients according to age and duration of CSCR is presented in Table 1.

**Table 1.** Distribution of patients according to age and duration of CSCR.

Age of patients in years	No	Percentage %
32 - 50	20	39.22
50 - 60	15	29.41
More than 60	16	31.37
Duration of CSCR in months	No	Percentage %
4 – 6	18	35.29
6 - 12	11	21.57
Longer than 12	22	43.14

None of the patients had previously undergone any invasive treatment due to CSCR, such as laser photocoagulation or photodynamic therapy; however, some of the patients were earlier treated by oral or topical non-steroidal drugs or troxerutin without therapeutic success. Prior to the treatment, all cases were subject to observation in other clinics. Diagnostic criterion of CSCR was the presence of subretinal fluid (SRF), sometimes accompanied by pigment epithelial detachment (PED), in the absence of choroidal neovascularization (CNV). CSCR was diagnosed by spectral optical coherence tomography (SOCT, Cirrus 4000, Zeiss, Germany 2012), fluorescein angiography, and fundus autofluorescence (FF-450, Zeiss, Germany 2010). The presence of SRF was determined by SOCT and FA. FAF was used to reveal changes in retinal pigment epithelium (RPE) typical for chronic CSCR, so-called gravitational tracks, places of lipofuscin accumulation in the RPE cells and RPE loss. Choroidal neovascularization was excluded by FA. Angio-OCT and indocyanine green angiography was not at our disposal during the study. Patients suspected of having CNV were excluded. All patients underwent a full ophthalmological examination, which included a review of their medical history and measurements of BCVA on a Snellen chart. For statistical analysis results were converted into log MAR scale. SOCT was used for determining the following parameters: central retinal thickness (CRT), central retinal volume (cube volume, CV), average central retinal thickness (CRTA) and maximum SRF height. In all cases SRF was found to be present under the foveola, however, in some eyes its maximum height was not located exactly at the center of the macula. Therefore, it deemed advisable to find additional parameter to measure the maximum change in retinal morphology after SMPLT.

BCVA and SOCT parameters were measured both prior to treatment and 2 months after each SMPLT. In cases where satisfactory results were not observed 2 months after the first SMPLT (residual subretinal fluid was still present), another SMPLT session was scheduled in 1 month’s time (hence a 3-month interval between the SMPLT sessions). BCVA and SOCT measurements were then taken once again 2 months after the second SMPLT. Analysis of the material was made after a maximum of two laser sessions. During the SMPLT procedure, the whole SRF area was covered with confluent foci of micropulse yellow laser (577nm) (Supra Scan 577, Quantel Medical, France 2014), based on the SOCT retinal maps. The focus diameter was set at 160 μm, power at a fixed level of 250mW, time of exposure at 0.2 s, and duty cycle at 5%.

### Statistical analysis

The primary measure of effectiveness of the SMPLT was percentage of patients with total resolution of subretinal fluid after maximum of two sessions of subthreshold micropulse laser. Secondary measures were decrease in the value of retinal parameters: CRT, CRTA, CV and SRF height and improvement of BCVA. Statistical analysis was conducted by Wilcoxon test.

For correlation between success of SMPLT (total resolution of SRF) and baseline patient characteristics, the values of different morphological and functional parameters before treatment were compared between the group of patients with total resolution of SRF and the group of eyes where SRF was still present after SMPLT. Difference in mean values of CRT, CRTA, CV, SRF height, BCVA, age of patient, and duration of CSCR between those two groups was measured. Analysis of statistical significance of the differences between two means was performed using U Mann-Whitney test.

Additionally, the influence of duration of CSCR on the potential morphological and functional improvement after SMPLT was also analyzed.

For that purpose, OCT measurements and BCVA were evaluated in the group of patients who responded well to SMPLT (total resolution of SRF) in the relation to the duration of CSCR. This group was divided into two subgroups: one with duration of CSCR up to 12 months (18 eyes) and the second with duration of CSCR above 12 months (33 eyes). One year seemed long enough to demonstrate eventual influence of the longstanding presence of subretinal fluid on retinal architecture. The mean results of the SOCT measurements (CRT, CRTA, CV, maximum fluid height) and BCVA after SMPLT were compared between those two subgroups. Statistical significance of the difference of two means was evaluated by U Mann-Whitney test and t-Student test.

Statistical analysis was performed using Statistica 10.0 (StatSoft Inc., 2011), and the following primary parameters of descriptive statistics were selected: arithmetic mean (M), median (Me), standard deviation (SD), first and third quartiles (Q1 and Q3) and the minimum (Min) and maximum (Max) values. The assumed distribution of variables compatibility with normal distribution were tested by the Shapiro-Wilk. The statistical hypotheses were verified using the t-Student test, Mann-Whitney U test and Wilcoxon test. The results were quantified statistically significant in cases where the calculated probability satisfied the inequality test, p <0.05.

## Results

The results of SPMLT are presented in Table 2. The change in all presented SOCT parameters and BCVA was statistically significant. The average values show a reduction of retinal edema as well as the volume of SRF. The improvement of visual acuity is not, however, spectacular at equivalent to approximately one line on the Snellen chart. The complete resorption of SRF was achieved in 36 cases (70.6%). In remaining 18 cases SRF was still present after two sessions of SMPLT. Among patients with complete resorption of SRF only five required second sessions of SMPLT (13,9%).

**Table 2.** Results of SMPLT (arithmetic mean values), Wilcoxon test.

Parameter	Before SMPLT	SD value	After SMPLT	SD value	Change	P value
CRT fovea μm	337.6	97.2	260.0	67.4	-77.6	0.0000
CV μm^3^	10.8	1.15	10.3	0.77	-0.59	0.0001
CRT average μm	301.0	32.1	286.9	21.3	-13.1	0.0001
SRF maximum height μm	178.5	109.3	48.2	115.4	-130.3	0.0000
BCVA (logMAR)	0.39	0.25	0.56	0.31	-0.08	0.0001

CRT central retinal thickness, CV retinal cube volume, BCVA best corrected visual acuity in decimal score, SD standard deviation

Table 3 presents the difference in baseline morphological and functional parameters between the group of patients with total resolution of SRF after SMPLT and the group of patients with SRF still present after the treatment. This chart presents also difference in age and duration of symptoms between these groups (Table 3).

**Table 3.** Statistical significance of differences between means of baseline morphological and functional parameters in the group of patients with total resolution of SRF after SMPLT and patients with SRF still present after SMPLT. (U Mann-Whitney test).

Parameter	Absence of SRF after SMPLT			SRF present after SMPLT			p
	M	Me	SD	M	Me	SD	
CRT(μm)	335.2	314	104.1	343.5	348	80.9	0.5558
SRF level (μm)	169.9	142	93.6	199.0	144	141.8	0.5149
CV (μm^3^)	11.0	10.7	1.2	10.5	10.5	1.0	0.4443
CRTA(μm)	304.9	297.5	33.2	291.9	293	28.2	0.3740
BCVA (logMar)	0.37	0.30	0.26	0.47	0.40	0.20	0.1210
Age (years)	51.5	49	11.0	59.3	54	9.9	0.0207
Duartion of CSCR (months)	20.1	13.5	17.7	14.3	12	17.1	0.1162

SMPLT subthreshold micropulse laser treatment, SRF subretinal fluid, M mean value, Me median , SD standard deviation, CRT central retinal thickness, CV cube volume, CRTA central retinal thickness average, BCVA best corrected visual acuity

A statistically significant correlation was found between the age of patients and their morphological response to SMPLT. Patients with complete resolution of subretinal fluid were significantly younger than patients with persistent SRF after SMPLT (accordingly M=51.5 years SD=11.0 and M=59.3 years SD=9.9). A correlation between the duration of CSCR and retinal thickness was also observed - a general cognition being that sensory retina gets thinner as CSCR progresses. This change in retinal morphology can be measured in patients with absolute resolution of SRF after SMPLT (Table 4).

**Table 4.** Retinal parameters and BCVA in patients with resolution of SRF after SMPLT (arithmetic mean values) in relation to the duration of CSCR. Statistical significance of the difference of two means (Student-t test and U Mann-Whitney test for BCVA only).

Parameter	Duration of CSCR up to 12 months	SD	Duration of CSCR longer than 12 months	SD	p value
CRT fovea μm	239.4	32.71	213.7	33.44	0.0257
CV μm^3^	10.5	1.38	9.9	0.85	0.0168
CRT average μm	290.8	38.36	275.6	23.90	0.0178
BCVA after SMPLT (logMAR)	0.28	0.20	0.34	0.24	0.6210

CRT central retinal thickness, CV cube volume, BCVA best corrected visual acuity, SD standard deviation, SMPLT subthreshold micropulse laser treatement

As can be observed from the data, all retinal parameters are lower in patients who had CSCR for a longer period of time however there is no statistically significant difference in BCVA.

## Discussion

SMPLT has been used intensively for the treatment of retinal edema of various origins during the last few years [35–37]. Clinical entities treated by SMPLT include diabetic macular edema (DME), CSCR, retinal edema secondary to retinal vein occlusion (RVO), or even exudative macular degeneration. The morphological results are usually promising. However, in a certain percentage of cases SMPLT does not have the expected effect, especially regarding improvement of BCVA. In the treatment of DME authors recommend the use of SMPLT for smaller edemas of less than 400 μm [38–41]. So far, recommendations for the use of SMPLT in CSCR are not unequivocal and it is not clear which patients could benefit the most from this treatment. Hence, the interest on our part in evaluating factors that could influence the outcome of this treatment. To our knowledge it is the first study to evaluate these factors in the same fashion, as it is usually done with other clinical entities, such as AMD or DME. Other authors have reported SMPLT efficacy in CSCR measured as reduction of retinal thickness and improvement of BCVA. Luttrull achieved the resolution of SRF after SMPLT in 11 cases of relatively short duration of CCSR (up to 7 months) [42]. Abd Elhamid reported a significant improvement in CRT and contrast sensitivity in 15 eyes with CSCR lasting 4.6 months on average [43]. Scholz et al. had morphological improvement rate of 74% in 38 cases of chronic CSCR including patients unresponsive to PDT [44]. Others reported short-term efficacy of SMPLT in reducing the CRT in relatively small groups of patients (10 to 15 eyes) with CSCR [45–47]. Özmert and Scholz compared SMPLT treatment to PDT in CSCR therapy showing better morphological results in laser treatment. [48, 49]

Table 5 presents number of eyes and type of treated cases in recent studies.

**Table 5.** Number of eyes and average duration of CSCR symptoms in recent studies. [39–46]

Study	No of eyes	Type of CSCR treated	Result CRT
Özmert E, J Ophthalmol. 2016	15	chronic	SRF resolution in 80%, BCVA improvement of 4.2 letters ETDRS
Scholz P, Eye 2016	42	chronic	CRT reduction in 79% of cases, mean values 445 μm to 297 μm
Luttrull JK, Retina 2016	11	acute/chronic	SRF resolution in all cases, mean BCVA improvement from 20/37 to 20/24
Abd Elhamid, Clin Ophthalmol. 2015	15	acute/chronic	CRT reduction from 389 μm to 263 μm Mean BCVA improvement from 0.67 to 0.85
Scholz P, Ophthalmologica 2015	38	chronic, including resistant to PDT	CRT reduction in 74%, mean CRT decrease 115 μm, BCVA improvement by logMAR-0.06
Kim JY, Graefes Arch Clin Exp Ophthalmol. 2015	10	chronic and recurrent	Mean BCVA improvement from 0.21 to 0.055 logMAR, mean CRT reduction from 349.2 μm to 250.7 μm
Yadav NK, Eye 2015	15	chronic	Mean BCVA improvement of one line on Snellen chart, 79% reduction in SRF height
Malik KJ, Retina 2015	11	acute/chronic	Mean 97 μm decrease of CRT
Present study	51	chronic	SRF resolution in 70.6 % of cases; mean BCVA improvement in logMAR 0.08

SRF subretinal fluid, CRT central retinal thickness, BCVA best corrected visual acuity

Nevertheless, it should be stressed that in some studies researchers treat both chronic and acute cases, which could have resolved spontaneously without any treatment. It is clear that establishing adequate criteria for chronicity of CSCR is important when evaluating the results of SMPLT. This study dealt with genuine chronic cases, the average duration of CSCR amounting to 18 months. Moreover, in comparison to other studies, this study is based on a reasonably large sample size, which allows the relevant statistical analysis to be carried out.

The study included patients with CSCR in the form of SRF, sometimes accompanied by PED. Cases of chronic pure PED without SRF, which could have been treated as chronic CSCR, were also initially included as part of the sample; however, poor response to SMPLT treatment in such cases (20% success rate) led to the conclusion that they were in fact a form of RPE degeneration, and not an active form of CSCR, hence their exclusion from this study.

Our study’s results show high efficacy of SMPLT in providing resolution of SRF in chronic CSCR. Complete resolution of subretinal fluid is in our opinion sensible criterion of morphological success of the procedure. Cases that show a just a reduction in SRF height after SMPLT, are in our opinion not resolved cases. The persistence of even small amounts of SRF in the long term might lead to progressive retinal thinning and the loss of photoreceptors. Such conclusions have been confirmed by the analysis of the group of patients who experienced the complete resolution of SRF after SMPLT. In cases of longer duration of CSCR, that is more than 12 months, a decrease in retinal morphological parameters, such as CRT or CV was observed.

Generally, SMPLT improved retinal morphology however the improvement noted in BCVA is small, what remains in consent with results of other studies. [44–46] Significant improvement in BCVA was noted in the studies that included acute cases, which probably improved more than the chronic ones [42, 43]. It might be true that cases of CSCR of duration longer than 4 months generally do not improve well functionally. Theoretically, a longer duration of symptoms should correlate with lower BCVA before and after treatment. Nevertheless, this was not found to be the case for the whole group in our study. Hence, major visual loss may occur in the first months following the onset of symptoms and may not progress in a linear way during the following months. The logical conclusion that can be drawn from this, is that SMPLT treatment should be considered sooner than 4 months after onset of CSCR, especially in cases that do not show tendency for spontaneous resolution.

The study was not successful in explaining why some eyes do not react to SMPLT, with no correlation between response to treatment and duration of CSCR, initial BCVA or initial CRT being found. It is reasonable to assume that the answer lies in the retinal and choroidal morphology; however, this is yet to be determined. CSCR is not a disease, which follows the same logic as other retinal vascular clinical entities, such as retinal vein occlusion or DME. The loss of vision associated with the disease is not linear and the response to the treatment does not resemble that of DME or RVO. Nevertheless, at present SMPLT should be taken into consideration as an option for treatment of acute CSCR that do not show tendency for SRF reduction.

It remains uncertain whether SMPLT can be used in the prevention of the recurrence of CSCR. However, in our study during the 9 months of follow up no recurrence of SRF was observed.

SMPLT has to be compared with other pulse type of lasers of shorter pulse duration such as selective retina therapy (SRT). The goal of this kind of treatment is to selectively damage the RPE and stimulate proliferation and migration of the adjacent RPE cells into the damage site. In consequence, due to rejuvenation of the diseased sites, metabolism of the RPE improves, enabling elimination of the intraretinal or subretinal fluid [50]. Elsner et al. treated 27 patients with active CSCR with pulsed double-Q-switched Nd-YLF prototype laser (lambda=527 nm, t=1.7 micros) achieving resolution of SRF in 85.2% of cases [51]. Framme et al. treated relatively small group of acute (10 eyes) and chronic (16 eyes) CSCR cases with the same type of laser, but shorter pulse duration [52]. Complete SRF resolution was noted in all acute cases after 3 months. In chronic cases one laser session brought complete SRF resolution in 19% of treated eyes; however, retreatment with higher pulse energy resulted in success in 83.3% of the remaining chronic CSCR cases. BCVA improvement in chronic cases was minor (from 71.6 to 72.8 letters ETDRS. Park tested the efficacy of SRT with automated real-time feedback-controlled dosimetry in 50 eyes with chronic CSCR [53]. SRF resolved completely in 74% at 3 months with mean BCVA improvement from 0.44 to 0.37 logMAR. Results of SRT in chronic CSCR are similar to those achieved by SMPLT in both retinal morphology and BCVA change. Application of SRT results in similar percentage of patients with complete SRF resolution and, same as in SMPLT, only minor BCVA improvement. With the advent of commercially available SRT lasers, this kind of therapy might be also considered an option in the treatment of chronic CSCR; however, its cost has to be taken into consideration.

## Conclusions

Subthreshold micropulse is an effective form of therapy in improving retinal morphology in chronic CSCR. Functional results of SMPLT in longstanding CSCR are poor. In order to achieve good visual results, SMPLT may be considered in acute cases showing no tendency for spontaneous improvement. The prolonged presence of SRF leads to retinal thinning and loss of visual acuity.

The explicit factors that determine the effects of SMPLT in CSCR are yet to be identified, and, in all probability, are awaiting discovery within the field of ocular morphology.
